# Clinical Significance of MPO-ANCA in Eosinophilic Granulomatosis With Polyangiitis: Experience From a Longitudinal Chinese Cohort

**DOI:** 10.3389/fimmu.2022.885198

**Published:** 2022-06-27

**Authors:** Suying Liu, Linna Han, Yanhui Liu, Jun Yang, Yu Zhang, Mengtao Li, Xinping Tian, Xiaofeng Zeng, Li Wang, Fengchun Zhang

**Affiliations:** ^1^ Department of Rheumatology and Clinical Immunology, Peking Union Medical College Hospital, Chinese Academy of Medical Sciences and Peking Union Medical College, The Ministry of Education Key Laboratory, National Clinical Research Center for Dermatologic and Immunologic Diseases, Beijing, China; ^2^ Department of Rheumatology and Clinical Immunology, the Third Affiliated Hospital of Chongqing Medical University, Chongqing, China; ^3^ Department of Rheumatology and Clinical Immunology, Affiliated Hospital of Chifeng University, Chifeng, China; ^4^ Division of Nephrology, Nanfang Hospital, Southern Medical University, Guangzhou, China

**Keywords:** myeloperoxidase, antineutrophil cytoplasmic antibody, eosinophilic granulomatosis with polyangiitis, stratification, renal involvement

## Abstract

**Objectives:**

The aim of this study is to investigate the clinical significance of myeloperoxidase (MPO)–antineutrophil cytoplasmic antibody (ANCA) on eosinophilic granulomatosis with polyangiitis (EGPA) from a longitudinal Chinese cohort.

**Methods:**

A total of 120 patients with EGPA were consecutively enrolled and followed up. Two patients with PR3 ANCA was excluded and our analysis focused on the 118 patients with EGPA. On the basis of MPO-ANCA status, baseline clinical manifestations, treatment, and outcomes were analyzed. Logistic regression analysis was performed to analyze the independently associated factors for renal involvement.

**Results:**

ANCA positivity was observed in 24.2% of patients with EGPA. Patients with MPO-ANCA accounted for 20.8%. Patients with positive MPO-ANCA had higher levels of erythrocyte sedimentation rate (ESR), C-reactive protein, Birmingham Vasculitis Activity Score (BVAS), higher ratios of fever, myalgia, renal involvement, and biopsy-proven vasculitis. Heart manifestations and asthma were more common in patients with negative ANCA. Baseline MPO-ANCA titers positively correlated with ESR, eosinophil count, and BVAS and were higher in patients with methylprednisolone pulse. Among patients with renal involvement, patients with positive MPO-ANCA had higher proportions of female, fever, biopsy-proven vasculitis, and faster ESR; patients with negative ANCA developed more skin and cardiac involvement. MPO-ANCA positivity, male, and ear involvement were the independent factors associated with renal involvement. Intravenous cyclophosphamide and immunoglobulins were prescribed more frequently in patients with positive MPO-ANCA.

**Conclusion:**

In this cohort, patients with positive MPO-ANCA and negative ANCA displayed distinct clinical features, suggesting that MPO-ANCA might be a valuable biomarker for EGPA stratification. Baseline MPO-ANCA level correlated positively with disease activity of EGPA. MPO-ANCA was a significant independent factor associated with renal involvement.

## Introduction

Eosinophilic granulomatosis with polyangiitis (EGPA), previously called Churg–Strauss syndrome, is a rare type of antineutrophil cytoplasmic antibody (ANCA)–associated systemic vasculitis (AAV) characterized by blood and tissue eosinophilia, necrotizing vasculitis, and granulomatous inflammation (1).Frequently, EGPA affects the respiratory tract, peripheral nervous system (PNS), cardiovascular system, kidney, gastrointestinal tract, and ENT (ear, nose, and throat) (2).

In the diagnosis of AAV, ANCA plays an important role, which displays perinuclear or cytoplasmic distribution on immunofluorescence with specificity against myeloperoxidase (MPO) or protease 3 (PR3) antigen (3). Although EGPA is a member of AAV, MPO-ANCA can only be detected in 30%–40% of patients with EGPA (4). The 1984 Lanham diagnosis criteria (5) and 1990 American College of Rheumatology criteria (6) do not include ANCA in the items, possibly due to the low detection rate. Previous studies from different cohorts suggest that ANCA is associated with clinical manifestations of EGPA (7–10). Large-scale retrospective studies on the relationship between EGPA and ANCA were conducted in Europe. However, among the Chinese population, the role of specific MPO-ANCA in the clinical spectrum of EGPA remains unclear. In this study, we aimed to investigate the significance of MPO-ANCA in the clinical characteristics, treatment, and outcomes of patients with EGPA from a longitudinal Chinese cohort.

## Patients and Methods

### Patients

A total of 120 patients with EGPA were identified by at least two rheumatologists in Peking Union Medical College Hospital (PUMCH) between January 2010 and December 2020. The patients were enrolled according to the 1990 American College of Rheumatology classification criteria for EGPA (6). Other causes of eosinophilia were carefully excluded from this cohort, such as parasite infection, eosinophilic pneumonia, eosinophilic gastroenteritis, asthma with hypereosinophilia, other allergic diseases, primary or neoplastic hypereosinophilic syndrome, and other neoplastic diseases. Written informed consent was obtained from all the patients. The ethical committee of PUMCH approved this study (approval number: S-K1385; Beijing, China). The study was performed in accordance with the ethical standards of the Declaration of Helsinki.

### ANCA Detection

The indirect immunofluorescence was used to test the cytoplastic ANCA (cANCA) and perinuclear ANCA (pANCA). Enzyme-linked immunosorbent assay or chemiluminescence was performed to identify the titer of target antigens, including MPO and PR3. ANCA was considered positive when the testing result using indirect immunofluorescence was positive, or the titer of MPO-ANCA and/or PR3-ANCA was higher than the reference value. ANCA was tested mainly at baseline, and continuous testing was lacking.

### Clinical Assessment at Baseline

Medical records were retrieved and reviewed to extract the clinical data, including the affected organs, laboratory testing, and histological findings. Organ involvement was assessed on the basis of medical history, laboratory tests, imaging, and biopsies. Each system involvement was defined on the basis of our previous descriptions with slight modifications (11). Asthma was defined as the active condition that required continuous glucocorticoids (GC; including inhaled, oral, or intravenous form) or with persistent dyspnea. Skin involvement included palpable purpura, reticulata, maculopapular rash, skin ulcers, or gangrene of the extremities. The definition of arthritis was swelling and pain in multiple joints with morning stiffness. Renal involvement was defined as abnormal urine test or serum creatinine beyond the upper limit of the normal range, and the former included hematuria, proteinuria (urine protein>0.5 g/24 h), and cylindruria. Gastrointestinal involvement included bleeding, obstruction, perforation of digestive tract, or abdominal symptoms that could not be explained by other disorders. PNS involvement was defined as mononeuritis multiplex and multiple peripheral neuropathy based on paresthesia or motion abnormality, electromyogram, and muscle biopsies. EGPA-associated central nervous system (CNS) involvement was diagnosed according to the clinical manifestations, physical examinations, and imaging, which included intracranial ischemia or hemorrhages, spinal cord or medulla oblongata involvement, and hypertrophic cranial pachymeningitis and precluded the CNS lesions caused by related risk factors, such as hypertension, hyperlipidemia, hyperglycemia, smoking, infection, and genetic factors. Cardiac involvement presented with myocardial involvement, heart failure, pericardial effusion, coronary lesions, moderate to severe valve involvement, and arrhythmia, which could not be explained by other reasons.

Biopsy findings were recorded as normal or showing evidence of eosinophilic infiltrates, vasculitis, and/or granuloma. Biopsy-proven vasculitis presented with inflammatory cell infiltration or fibrinoid necrosis in the wall of vessels, thickening of vessel walls, and/or internal elastic lamina rupture. The original 1994 Birmingham Vasculitis Activity Score (BVAS) was used to assess the disease activity at diagnosis (12). Disease features were only scored if they were attributable to active vasculitis. The prognosis was assessed according to the 2011 revised five factor score (FFS), which includes age over 65 years, cardiac symptoms, gastrointestinal involvement, renal insufficiency (peak serum creatinine ≥150 μmol/L), and the absence of ENT manifestations (13).

### Treatment Strategy

Patients were treated with therapy that was considered the most appropriate regimen at that time. The drugs used to induce and maintain remission were recorded in detail. For GCs, initial pulse of methylprednisolone (MP) was defined as 0.5~1.0 g/d for 3~5 days, high-dose prednisone was 1~2 mg kg^−1^ d^−1^, and medium-dose prednisone was 0.5~0.8 mg kg^−1^ d^−1^.

### Follow-Up and Outcomes

Long-term follow-up and outcomes were established after the patient’s last visit or death. Complete remission was achieved if the BVAS became zero, and partial relief was defined as a ≥50% decrease of BVAS from baseline for at least six months. Mortality analysis was based on all-cause mortality.

### Statistical Analysis

Clinical characteristics of patients are presented as numbers and percentages for categorical variables and as mean ± standard deviation (SD) or median (first and third quartiles) for continuous variables. All analysis was performed using SPSS 25.0. Differences between the patients with positive MPO-ANCA and negative ANCA regarding the continuous variables were tested using t-test or Mann–Whitney *U-*test, and differences in categorical variables were assessed with Fisher’s exact or Chi-square tests, as appropriate. Two-sided *P-*values of <0.05 were considered statistically significant. Survival analysis was conducted using Kaplan–Meier survival curves and log-rank tests (Prism 7; GraphPad, San Diego, CA, USA). Univariate and multivariate logistic regression analyses were performed to find out the independently associated factors for renal involvement in EGPA. Correlations were calculated using Pearson correlation analysis.

## Results

### Clinical Features of Patients With EGPA With MPO-ANCA and Without ANCA at Baseline

A total of 120 patients with EGPA were enrolled in our cohort. Positive ANCA was observed in 29 patients (24.2%), including 25 with MPO-ANCA (20.8%), 5 with PR3-ANCA (4.2%), 25 with pANCA (20.8%), and 7 with cANCA (5.8%). Three patients showed quadruple positive for MPO-ANCA, PR3-ANCA, pANCA, and cANCA.

We first explored the association between MPO-ANCA and the clinical spectrum in this cohort (**Table 1**). We removed two patients with only PR3-ANCA to ensure the data clarity. Patients with MPO-ANCA were relatively older than those without ANCA (50.6 ± 13.8 vs. 44.0 ± 15.0, p = 0.05), and the gender was not significantly different. Laboratory tests revealed that ESR [53 (40, 79) vs. 26 (9, 44) mm/1 h, p < 0.0001] and CRP [36.5 (22.4, 70.0) vs. 13.8 (5.2, 55.7) mg/L, p = 0.02] were significantly higher in the MPO-ANCA–positive group than in the ANCA-negative group. The eosinophil count and ratio were comparable between groups. Compared with the ANCA-negative group, the MPO-ANCA–positive group had higher percentages of patients with fever (84.0% vs. 40.9%, p = 0.0001) and myalgia (40.0% vs. 17.2%, p = 0.02). In terms of the systemic manifestations, renal involvement occurred more frequently in the MPO-ANCA–positive group (80.0% vs. 35.5%, p < 0.0001), whereas cardiac lesions (38.7% vs. 12.0%, p = 0.01) and asthma (76.3% vs. 56.0%, p = 0.04) presented more commonly in the ANCA-negative group. A total of 64.0% of patients with EGPA with MPO-ANCA developed PNS involvement compared with 43.0% in the ANCA-negative group, which was close to the statistical significance (p = 0.06).

**Table 1:** Clinical features of patients with EGPA with MPO-ANCA and without ANCA at baseline.

Characteristics	All patientsn = 118^a^	MPO-ANCA–positive, n = 25	ANCA-negative, n = 93	*P*-value
Age, years, mean ± SD	45.4 ± 15.0	50.6 ± 13.8	44.0 ± 15.0	**0.05**
Gender, male/female, number	69/49	13/12	56/37	0.46
Time from allergy to EGPA diagnosis (month), median (IQR)	31 (0, 77)	48 (7,72)	28 (0, 73)	0.50
Time from initial symptoms to EGPA diagnosis (month), median (IQR)	18 (3,53)	13 (6, 56)	18 (3,50)	0.83
Eosinophil count, median (IQR)	3.2 (1.5, 8.5)	4.2 (1.3, 8.7)	3.2 (1.6, 7.2)	0.72
Eosinophil ratio, median (IQR)	28.6 (16.9, 45.9)	31.9 (13.4, 44.1)	28.5 (18.3, 45.8)	0.91
ESR, mm/1 h, median (IQR)	34 (11, 53)	53 (40, 79)	26 (9, 44)	**<0.0001**
CRP, mg/L, median (IQR)	21.3 (6.1, 64.9)	36.5 (22.4, 70.0)	13.8 (5.2, 55.7)	**0.02**
RF, IU/ml, median (IQR)	15 (7, 72)	39 (6, 180)	13 (8, 58)	0.32
Eosinophilic infiltration, n (%)	52/75 (69.3)	10/13 (76.9)	42/62 (67.7)	0.74
Biopsy-proven vasculitis, n (%)	18/75 (24.0)	7/13 (53.8)	11/62 (17.7)	**0.01**
Granuloma, n (%)	11/75 (14.7)	3/13 (23.1)	8/62 (12.9)	0.39
**Clinical manifestations, n (%)**				
Fever	59 (50.0)	21 (84.0)	38 (40.9)	**0.0001**
Weight loss	45 (38.1)	12 (48.0)	33 (35.5)	0.26
Arthritis	17 (14.4)	3 (12.0)	14 (15.1)	1.00
Myalgia	26 (22.0)	10 (40.0)	16 (17.2)	**0.02**
Allergic rhinitis	50 (42.4)	8 (32.0)	42 (45.2)	0.23
Asthma	85 (72.0)	14 (56.0)	71 (76.3)	**0.04**
Gastrointestinal involvement	38 (32.2)	6 (24.0)	32 (34.4)	0.32
Renal involvement	53 (44.9)	20 (80.0)	33 (35.5)	**<0.0001**
Abnormal urine test	51 (43.2)	19 (76)	32 (34.4)	**0.0002**
Increased serum creatinine	15 (12.7)	8 (32.0)	7 (7.5)	**0.003**
Skin involvement	59 (50.0)	9 (36.0)	50 (53.8)	0.11
PNS involvement	56 (47.5)	16 (64.0)	40 (43.0)	0.06
CNS involvement	18 (15.4)	4 (16.0)	14 (15.2)	1.00
Cardiac involvement	39 (33.1)	3 (12.0)	36 (38.7)	**0.01**
Ear involvement	15 (12.7)	4 (16.0)	11 (11.8)	0.52
Sinusitis	72 (61.0)	12 (48.0)	60 (64.5)	0.13
Thrombotic event	25 (21.2)	5 (20)	20 (21.5)	0.87
BVAS>15	61 (51.7)	19 (76.0)	42 (45.2)	**0.006**
BVAS, median (IQR)	16 (10, 22)	21 (17, 24)	15 (10, 20)	**0.004**
Five factor score				0.35
0	29 (24.6)	7 (28.0)	22 (23.7)	–
1	49 (41.5)	11 (44.0)	38 (40.9)	–
2	28 (23.7)	3 (12.0)	25 (26.9)	–
3	12 (10.2)	4 (16.0)	8 (8.6)	–

a Two patients with only PR3-ANCA positivity were removed. ANCA, antineutrophil cytoplasmic antibody; BVAS, Birmingham Vasculitis Activity Score; CNS, central nervous system; CRP, C-reactive protein; EGPA, eosinophilic granulomatosis with polyangiitis; ESR, erythrocyte sedimentation rate; IQR, interquartile range; MPO, myeloperoxidase; PNS, peripheral nervous system; RF, rheumatoid factor. The bold values indicate statistically significant differences.

The disease activity and prognosis were also assessed for the patients. BVAS in patients with positive MPO-ANCA was higher than that in controls [21 (17, 24) vs. 15 (10, 20), p = 0.004], and the MPO-ANCA–positive group had a higher percentage of patients with active disease condition (76.0% vs. 45.2%, p = 0.006), which was defined as BVAS > 15. Overall FFS was similar between the groups, although the ratio of FFS = 3 in the MPO-ANCA–positive group was relatively higher than that in the control group (16.0% vs. 8.6%).

### Correlation of MPO-ANCA Titers at Onset and Other Parameters

**Figure 1:**
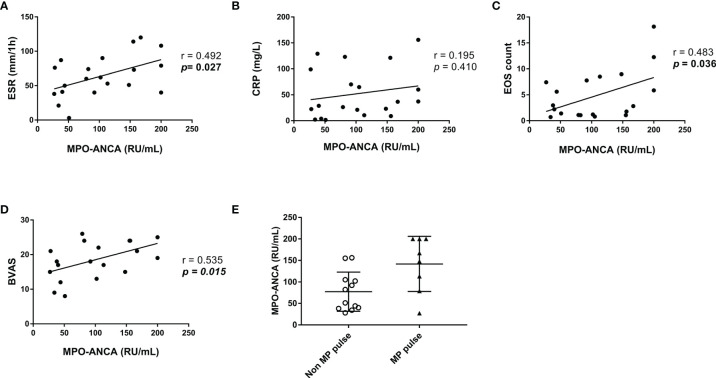
Correlation of MPO-ANCA titers at baseline with other parameters in the patients with MPO-ANCA-positive EGPA. **(A–D)** Correlation of MPO-ANCA titers with ESR, CRP, EOS count, and BVAS. Pearson correlation was used for the analysis. **(E)** Comparison of MPO-ANCA titers between patients with and without MP pulse. ANCA, antineutrophil cytoplasmic antibody; BVAS, Birmingham Vasculitis Activity Score; CRP, C-reactive protein; EGPA, eosinophilic granulomatosis with polyangiitis; EOS, eosinophil; ESR, erythrocyte sedimentation rate; MP, methylprednisolone; MPO, myeloperoxidase.

Among the MPO-ANCA–positive patients with EGPA at baseline, we analyzed the correlation of MPO-ANCA titers and ESR, CRP, eosinophil count, and BVAS (**Figures 1A–D**). The results suggested that MPO-ANCA titers positively correlated with ESR (r = 0.492, p = 0.027 eosinophil count (r = 0.483, p = 0.036), and BVAS (r = 0.535, p = 0.015). Moreover, the patients with EGPA who were given MP pulse had higher MPO-ANCA titers at onset than those who never experienced MP pulse (**Figure 1E**).

### Relationship Between Renal Involvement and MPO-ANCA

The analysis above emphasized that the ratio of renal involvement in the MPO-ANCA–positive group was significantly higher compared with the ANCA-negative group. However, a large proportion of patients with renal lesions were ANCA-negative (33 of 53). Therefore, we further investigated the differences between patients with postive MPO-ANCA and negative ANCA with renal lesions **(** **Table 2** **)**. The results indicated that female percentage was higher in the MPO-ANCA–positive group than in the ANCA-negative group (45% vs. 18%, p = 0.04). ESR was faster in the MPO-ANCA–positive group compared with the negative group [61 (40, 85) vs. 24 (10, 45) mm/1 h, p < 0.001]. The biopsy-proven vasculitis (44.4% vs. 8.7%, p = 0.04) and fever (85.0% vs. 42.4%, p = 0.002) in patients with MPO-ANCA were more common than in patients without ANCA. On the other hand, the patients with negative ANCA with renal involvement had higher percentages of skin lesions (60.6% vs. 25.0%, p = 0.01) and cardiac involvement (42.4% vs. 15.0%, p = 0.04). Gastrointestinal involvement showed a higher tendency in the patients with negative ANCA with renal involvement (39.4% vs. 15.0%, p = 0.06).

**Table 2:** Clinical characteristics of patients with EGPA with renal involvement at baseline.

Characteristics	MPO-ANCA–positive, n = 20	ANCA-negative, n = 33	*P*-value
**Demographics**			
Age, years, mean ± SD	51.3 ± 14.5	45.3 ± 15.3	0.17
Gender, male/female, number	11/9	27/6	**0.04**
Time from allergy to EGPA diagnosis (month), median (IQR)	48 (12, 72)	6 (0, 60)	0.16
Time from initial symptoms to EGPA diagnosis (month), median (IQR)	13 (5, 54)	12 (2, 55)	0.90
**Laboratory tests**			
Eosinophil count, median (IQR)	3.0 (1.3, 7.6)	3.6 (2.4, 8.3)	0.62
Eosinophil ratio, median (IQR)	28.5 (12.2, 40.8)	30.9 (19.7, 45.8)	0.45
ESR, mm/1 h, median (IQR)	61 (40, 85)	24 (10, 45)	**<0.001**
CRP, mg/L, median (IQR)	36.8 (24.4, 84.4)	19.6 (7.2, 81.0)	0.13
RF, IU/ml, median (IQR)	39 (16, 135)	14 (10, 51)	0.35
Extravascular eosinophilic infiltration, n (%)	7/9 (77.8)	16/23 (69.6)	1.00
Biopsy-proven vasculitis, n (%)	4/9 (44.4)	2/23 (8.7)	**0.04**
Granuloma, n (%)	2/9 (22.2)	2/23 (8.7)	0.56
**Clinical manifestations, n (%)**			
Fever	17 (85.0)	14 (42.4)	**0.002**
Weight loss	10 (50.0)	14 (42.4)	0.59
Arthritis	2 (10.0)	6 (18.2)	0.70
Myalgia	8 (40.0)	7 (21.2)	0.14
Allergic rhinitis	6 (30.0)	12 (36.4)	0.64
Asthma	11 (55.0)	23 (69.7)	0.28
Gastrointestinal involvement	3 (15.0)	13 (39.4)	0.06
Skin involvement	5 (25.0)	20 (60.6)	**0.01**
PNS involvement	13 (65.0)	15 (45.5)	0.17
CNS involvement	3 (15.0)	8 (24.2)	0.50
Cardiac involvement	3 (15.0)	14 (42.4)	**0.04**
Ear involvement	4 (20.0)	7 (21.2)	1.00
Sinusitis	11 (55.0)	23 (69.7)	0.28
Thrombotic event	3 (15.0)	10 (30.3)	0.33
BVAS>15	17 (85.0)	21 (63.6)	0.09
BVAS, median (IQR)	22 (18, 25)	18 (13, 28)	0.50
Five factor score, n (%)			0.37
0	6 (30.0)	7 (21.2)	–
1	9 (45.0)	13 (39.4)	–
2	1 (5.0)	8 (24.2)	–
3	4 (20.0)	5 (15.2)	–

ANCA, antineutrophil cytoplasmic antibody; BVAS, Birmingham Vasculitis Activity Score; CNS, central nervous system; CRP, C-reactive protein; EGPA, eosinophilic granulomatosis with polyangiitis; ESR, erythrocyte sedimentation rate; IQR, interquartile range; MPO, myeloperoxidase; PNS, peripheral nervous system; RF, rheumatoid factor. The bold values mean statistical differences.

**Figure 2:**
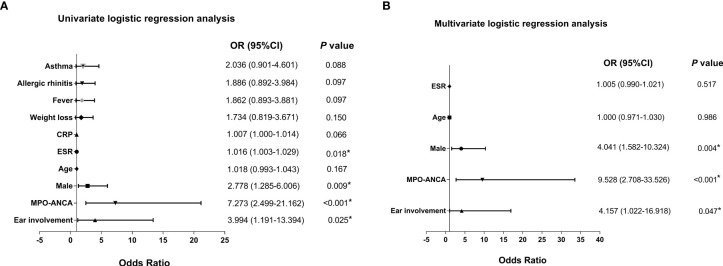
Forest plots of univariate and multivariate logistic regression analysis of renal involvement of patients with EGPA. Forest plots of **(A)** univariate and **(B)** multivariate logistic regression analysis of patients with EGPA with renal involvement. ANCA, antineutrophil cytoplasmic antibody; CRP, C-reactive protein; EGPA, eosinophilic granulomatosis with polyangiitis; ESR, erythrocyte sedimentation rate; MPO, myeloperoxidase; OR, odds ratio. *P < 0.05.

We further analyzed the independent factors associated with renal lesions in EGPA. We first screened out the possible related factors through univariate logistic regression analysis (**Figure 2A**). Then, after adjustment for age using multivariate logistic regression analysis, three independent associated factors were finally revealed which were male (OR = 4.041, 95% CI: 1.582–10.324, p = 0.004), ear involvement (OR = 4.157, 95% CI: 1.022–16.918, p = 0.047), and MPO-ANCA positivity (OR = 9.528, 95% CI: 2.708–33.526, p < 0.001) (**Figure 2B**).

### Pathologic Characteristics

A total of 77 patients underwent one or more biopsies from different tissues or organs. Extravascular eosinophilic infiltration was present in 70.1% (54 of 77) of the patients, but there was no difference between the groups. Biopsy-proven vasculitis was seen in 23.4% (18 of 77) of all the patients with EGPA and frequently occurred in kidneys and peripheral nerves. Granulomas were relatively rare (14.3%, 11 of 77), occasionally observed in lungs and skin. Detailed records of renal pathology were from three patients and all of them were males. Two of the patients were MPO-ANCA–positive, which both showed crescentic glomerulonephritis, accompanied by glomerular necrosis in different ranges without obvious immune complex deposition. Another patient was MPO-ANCA–negative and developed mesangial proliferative glomerulonephritis. In this study, the patients with positive MPO-ANCA showed a significantly higher ratio of biopsy-proven vasculitis than the patients with negative ANCA (53.8% vs. 17.7%, p = 0.01; **Table 1**), but eosinophilic infiltration and granulomas were both comparable between the groups.

### Treatment

**Figure 3:**
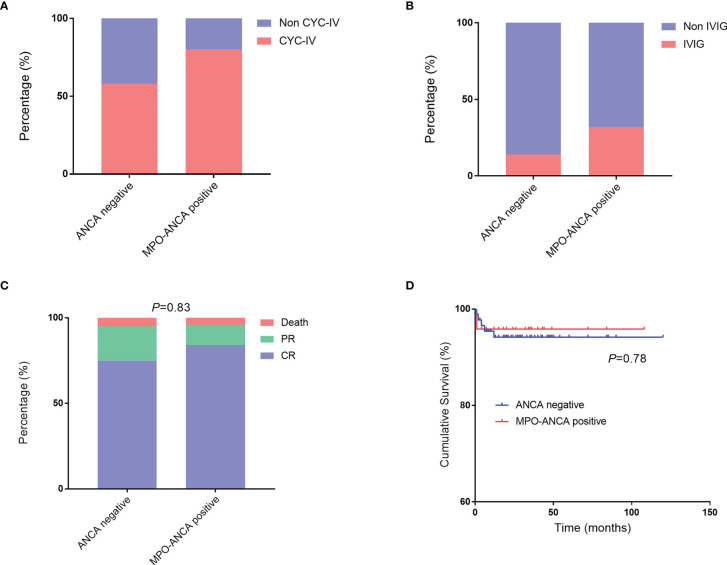
Treatment and outcomes in patients with EGPA with MPO-ANCA and without ANCA. **(A, B)** Comparisons of CYC-IV and IVIG used in patients with MPO-ANCA and without ANCA. **(C, D)** Comparisons of outcomes and cumulative survival rates between the two groups. ANCA, antineutrophil cytoplasmic antibody; CYC-IV, intravenous cyclophosphamide; EGPA, eosinophilic granulomatosis with polyangiitis; IVIG, intravenous immunoglobulin; MPO, myeloperoxidase.

The GC therapy was administered to 117 patients, and three patients selected other therapies because their conditions were relatively good and they were too concerned about the adverse effects of GC. MP pulse was prescribed to 36.0% (nine) of patients with positive MPO-ANCA and 24.7% (twenty-three) of patients with negative ANCA, which did not reach the statistical difference. Intravenous cyclophosphamide (CYC) was more commonly administered in the MPO-ANCA–positive group than in the ANCA-negative group (80.0% vs. 58.1%; **Figure 3A**).

Patients with positive MPO-ANCA were used a relatively higher ratio of intravenous immunoglobulin (IVIG) therapy than the patients with negative ANCA (32.0% vs. 14.0%; **Figure 3B**). We retrospectively analyzed the outcomes between patients who had used IVIG at least once and those who had never used IVIG (**Supplementary Table 1**). The result showed that all death cases occurred in the group without IVIG therapy. The details of patients who had used IVIG were presented in **Supplementary Table 2**. A total of 21 patients with EGPA were administered IVIG, most of whom were prescribed IVIG together with GC and CYC for inducing remission. IVIG was generally prescribed 20 g per day for 3–5 days. Most of the patients who used IVIG therapy had FFS of ≥1 (18 of 21) or had cardiac manifestations or peripheral neuropathy (17 of 21). The median of BVAS for them was 18. Finally, they got complete remission (17 of 21) or partial relief (4 of 21) under the treatment strategy.

### Outcomes

The median follow-up duration was 26 months (range: 1–120 months). Of all the patients with EGPA, 77.3% and 17.6% achieved complete remission and partial relief, respectively. The overall outcomes were not significantly different between the two groups (**Figure 3C**). We also summarized the clinical features of death cases in this cohort (**Supplementary Table 3**). Six patients died, including one with MPO-ANCA (Case 1, 4.0%) and five without ANCA (5.4%). Among them, two patients died due to cancers which were chronic myelomonocytic leukemia and lung cancer; one was due to digestive tract perforation; one was diagnosed as intestinal perforation combined with infection and myocardial involvement; one died of multiple organ failure combined with severe infection; and the last one died of a sudden aneurysm rupture. Of note, most of the death events (five of six) occurred within 6 months, and most patients had used GC and CYC (only one patient used hydroxychloroquine and tripterygium) but none had tried IVIG.

We further performed the survival analysis and found that the cumulative survival rates were comparable in the two groups (**Figure 3D**).

## Discussion

The critical role of ANCA in AAV remains to be a focus. Many studies have explored it extensively, mainly in GPA and MPA. In EGPA, the clinical significance of specific MPO-ANCA, particularly in Chinese patients, was elusive. In this study, we retrospectively investigated the impact of MPO-ANCA on EGPA. The patients with positive MPO-ANCA had higher disease activity and rates of renal involvement, biopsy-proven vasculitis, fever, and myalgia. The patients with negative ANCA developed more cardiac involvement and asthma. The baseline MPO-ANCA titers correlated positively with disease activity. MPO-ANCA was the most significant independent factor associated with renal involvement. Patients with positive MPO-ANCA were administered intravenous CYC and IVIG more frequently than patients with negative ANCA.

In the published papers about EGPA, patients were almost from the cohort of the French Vasculitis Study Group or the United States (7, 8, 14–19), and comprehensive data from Asia, especially from China, were scarce. Therefore, we established such an EGPA cohort to provide more information from China in the field of EGPA. The French Vasculitis Study Group published their findings 20 years ago (14) and subsequently updated many contents from the same cohort in 2013 and 2021 (8, 17), which may be because the disease process and outcomes have been changed with the development of EGPA diagnosis and treatment. For instance, one study from Rodriguez-Pla et al. revealed that, since 1999, the mortality rates of patients with primary systemic vasculitis have progressively decreased in the United States, and they demonstrated the mortality trends of vasculitis in gender, racial, and geographic disparities (19). Therefore, studies from different regions, races, and year ranges are warranted.

We described some similar results with publications of Sablé-Fourtassou et al. or Sinico et al. from European EGPA cohorts (14, 15). However, our study had distinct findings, which provided more evidence for the clinical significance of MPO-ANCA. Notably, ANCA includes MPO-ANCA, PR3-ANCA, and ANCAs without specificity according to target autoantigens. The French cohort in 2013 reported that only 63% of ANCA positivity was positive for MPO-ANCA (8). Many previous studies have just selected total ANCAs detected by immunofluorescence as the stratification marker (7–9). However, our preliminary analysis found that the clinical phenotypic differences of EGPA were more significant based on MPO-ANCA grouping than ANCA grouping. Therefore, this study specifically explored the clinical significance of MPO-ANCA. We compared two European EGPA cohorts with ours as a representative Asian cohort and found some disparities (**Table 3**). First, our study indicated that fever was more common, and the proportion of fever and myalgia in the MPO-ANCA–positive group was significantly higher than that in the ANCA-negative group. Second, the percentage of asthma at baseline was relatively lower in our cohort, and the ANCA-negative group had a higher proportion of asthma than the MPO-ANCA–positive group. Therefore, it should be aware that not all patients with EGPA would develop asthma in clinical practice especially for the respiratory physician. Third, we demonstrated that BVAS, ESR, and CRP in MPO-ANCA–positive group were significantly higher, and baseline MPO-ANCA titers positively correlated with ESR, eosinophil count, and BVAS, implying the possible pathogenic role of MPO-ANCA and the necessity of repeated MPO-ANCA detection. Moreover, EGPA-associated CNS involvement occurred more commonly in our cohort. Regarding the prognosis and treatment, the European cohort from Papo et al. had a higher proportion of patients with FFS of ≥2 in the MPO-ANCA–positive group than the control group, and MP pulse and immunosuppressants for induction were used more in the patients with positive MPO-ANCA (17). Concerning the mortality, the 2013 French cohort grouped by ANCA revealed that the ANCA-negative group had a higher mortality than the ANCA-positive group at 10 years (8). We found that the proportion of FFS of ≥2 in ANCA-negative group was higher (ANCA^+^ vs. ANCA^−^: 33% vs. 46%), but the usage percentage of immunosuppressants for induction was comparable between the two groups and the total usage ratio of immunosuppressants was significantly lower than that in ours and the study of Papo et al. Therefore, we speculated that, at that time, the treatment might be inadequate for patients with negative ANCA in this early EGPA cohort. Together, this cohort from China might reflect some discrepancies between different regions, ethnicities, and year ranges in EGPA.

**Table 3:** Comparison of EGPA cohorts from different regions.

	Our study, 2022	Papo et al., 2021 (17)	Comarmond et al., 2013^a^ (8)
Country/Region	China	European	France
Patients number	120	734	348
ANCA+, n (%)	29 (24.2)	226 (30.8)	108 (31.0)
MPO-ANCA+, n (%)	25 (20.8)	210 (28.6)	68 (19.5)
PR3-ANCA+, n (%)	5 (4.2)	16 (2.2)	4 (1.1)
**MPO-ANCA+ vs. ANCA-**			
Female gender, %	48 vs. 40	48 vs. 58	44 vs. 48
Age, years, mean	**51 vs. 44**	**57 vs. 51**	53 vs. 50
CRP, median, mg/L	**37 vs. 14**	**66 vs. 18**	**79 vs. 59**
Eosinophils, median,/mm^3^	4.2 vs. 3.2	5.4 vs. 3.2	7.8 vs. 7.3
Fever, %	**84 vs. 41**	36 vs 27	41 vs. 35
Myalgia, %	**40 vs. 17**	31 vs. 28	41 vs. 38
Arthralgia, %	12 vs. 15	38 vs. 25	34 vs. 27
Asthma, %	**56 vs. 76**	91 vs. 93	93 vs. 91
Skin involvement, %	36 vs. 54	38 vs. 34	45 vs. 36
PNS involvement, %	64 vs. 43	**71 vs. 47**	**63 vs. 44**
CNS involvement, %	16 vs. 15	5 vs. 3	7 vs. 4
Renal involvement, %	**80 vs. 36**	**29 vs. 5**	**27 vs. 16**
Gastrointestinal involvement, %	24 vs. 34	15 vs. 16	22 vs. 23
Cardiac involvement, %	**12 vs. 39**	**21 vs. 33**	**8 vs. 19** ^b^
ENT manifestations, %	60 vs. 65	84 vs. 83	**59 vs. 44**
BVAS, median	**21 vs. 15**	**16 vs. 13**	**21 vs. 18**
Five factor score, %			
FFS = 0	28 vs. 24	59 vs. 63^c^	33 vs. 22
FFS = 1	44 vs. 41	22 vs. 30^c^	34 vs. 32
FFS ≥ 2	28 vs. 35	**19 vs. 7** ^c^	33 vs. 46
Pulses of methylprednisolone, %	36 vs. 25	**58 vs. 46**	——
Immunosuppressant for induction, %	92 vs. 85	**72 vs. 62**	50 vs. 56
Death, %	4 vs. 5	6 vs. 5	**6 vs. 13**

^a^This cohort study was based on ANCA grouping, not MPO-ANCA. ^b^Only cardiomyopathy was included in this analysis. ^c^The 1996 Five Factor Score system was used here. Numbers shown in bold are statistically significant. ANCA, antineutrophil cytoplasmic antibody; BVAS, Birmingham Vasculitis Activity Score; CNS, central nervous system; CRP, C-reactive protein; EGPA, eosinophilic granulomatosis with polyangiitis; ENT, ear, nose, throat; FFS, five factor score; MPO, myeloperoxidase; PNS, peripheral nervous system; PR3, protease 3.

MPO-ANCA can activate neutrophils in many ways, releasing reactive oxygen species, granule proteins, and cytokines, which damages tissues (20). In our study, the patients with positive MPO-ANCA suffered more fever and myalgia and had higher ESR, CRP, and BVAS, suggesting MPO-ANCA as an inflammatory mediator. The renal histology implied pauci-immune necrotizing crescentic glomerulonephritis in the patients with positive MPO-ANCA. A recent study with 63-biopsy-proven renal involvement also revealed necrotizing pauci-immune glomerulonephritis as the most common presentation in ANCA-positive patients with EGPA (21). Animal studies have confirmed that the anti-MPO antibody alone can cause necrotizing crescentic glomerulonephritis (22, 23). Together, MPO-ANCA plays an essential role in the pathogenesis of EGPA.

We further explored the correlation of MPO-ANCA level with disease activity in EGPA. The analysis was based on baseline ANCA testing results because repeat ANCA testing was not a routine in our hospital. For patients with positive MPO-ANCA at baseline, MPO-ANCA titers positively correlated with parameters suggestive of disease activity and were associated with the ratio of MP pulse in this cohort. A study from Mayo Clinic reported that 75% of patients with EGPA were ANCA positive during the disease flare, whereas only 16% during remission. ANCA prevalence was 73% before and 36% after treatment. Serial measurements indicated a correlation of MPO-ANCA titers with disease status (24). A prospective AAV cohort study from Japan in 2018 revealed that reappearance of MPO-ANCA may be useful for predicting relapse in the patients with MPO-ANCA–positive AAV in remission. MPO-ANCA could convert to negative after treatment (25). Therefore, for patients who were positive for MPO-ANCA at baseline, monitoring MPO-ANCA levels may be meaningful in terms of observing disease activity and predicting relapse.

Whether EGPA can be divided into two subtypes based on the ANCA status remains controversial. According to our cohort, the MPO-ANCA–positive group had more severe inflammation, higher disease activity and frequencies of renal involvement and biopsy-proven vasculitis, and the ANCA-negative group developed more cardiac manifestations and asthma. The large European cohort reported some similar findings (17). Conversely, the 2020 international consensus on ANCA testing in EGPA suggested that the MPO-ANCA is neither sensitive nor specific enough to identify “vasculitic” or “eosinophilic” EGPA, although patients with MPO-ANCA have more features of vasculitis (26). Even so, it did not mean that MPO-ANCA is not suitable for subclassification, if not simply limited to classify as “vasculitic” and “eosinophilic”. Furthermore, a recent genome-wide association study of EGPA stratified by MPO-ANCA suggests that EGPA may comprise two clinically and genetically distinct syndromes. MPO+ EGPA shares a strong HLA-DQ association with anti-MPO AAV, whereas ANCA-negative EGPA presents with mucosal/barrier dysfunction. Variants with IRF1/IL5 are associated with ANCA-negative EGPA, suggesting that anti-IL5 therapy might be more specific for this subset (27). Although functional studies are needed, this study suggests that patients with positive MPO-ANCA and negative ANCA have fundamentally different genetic background in pathogenesis, which supports MPO-ANCA as a potential biomarker of EGPA stratification.

More specifically, a recent study investigated the clinicopathologic features of EGPA-associated neuropathy with and without MPO-ANCA (10). Compared with the ANCA-negative group, the MPO-ANCA–positive group had higher CRP and percentage of vasculitis suggested by sural nerve biopsy but lower eosinophil infiltration in affected tissues. Furthermore, rare PR3+ EGPA also showed distinct features compared with patients with positive MPO-ANCA and negative ANCA. French Vasculitis and EGPA European Study Group in 2021 reported that PR3+ patients with EGPA shared more clinical features with GPA. Compared with patients with positive MPO-ANCA and negative ANCA, patients with PR3+ ANCA less frequently developed active asthma and peripheral neuropathy and more frequently had cutaneous manifestations and pulmonary nodules and lower eosinophil count (17). Taken together, MPO-ANCA may be an effective stratification biomarker for EGPA.

Recently, the newly revised classification criteria of AAV have incorporated the MPO-ANCA and PR3-ANCA into the system for the first time, highlighting the significance of specific ANCA (28–30). Positive cANCA or PR3-ANCA in GPA and pANCA or MPO-ANCA in MPA are weighted largely in the new criteria, because these antibodies are very commonly seen in GPA or MPA. In EGPA, cANCA or PR3-ANCA is a deduction item, and pANCA or MPO-ANCA is not included in the criteria, possibly because MPO-ANCA positivity is relatively low in patients with EGPA. Therefore, MPO-ANCA may be not appropriate for EGPA classification, but be meaningful for subtype stratification as MPO-ANCA–positive, PR3-ANCA–positive, and ANCA-negative EGPA.

The therapy of CYC combined with GC induced long-term remission in more than 90% of patients with AAV (31), which was the core treatment in our cohort. IVIG was more commonly prescribed in the MPO-ANCA–positive group. More importantly, we found that for patients who were ever administered IVIG therapy, the outcomes might be better, in particular, all deaths occurred in the group without IVIG therapy. Most of the death cases were recorded within 6 months. Therefore, early combination with IVIG on basis of GC and CYC might yield better outcomes for these patients. However, this result is only a rough analysis with confounding factors because it was based on a retrospective cohort. Interestingly, we noted that IVIG is a mainstay in the treatment of Kawasaki disease which is a form of acute vasculitis that mainly occurred in children. If not intervened in the early stage, about 25% of patients will develop serious coronary artery aneurysms (32, 33). Newburger et al. in 2016 reviewed that the risk of coronary artery aneurysms was reduced five-fold if IVIG was used within 10 days of fever onset (34). In our cohort, one patient with EGPA died from a sudden aneurysm rupture, implying the potential benefit of early IVIG usage to prevent serious artery aneurysms in EGPA. A meta-analysis suggested that IVIG potentially reduced levels of ANCA, BVAS, and CRP in patients with active AAV (35). In theory, IVIG preparations might be implicated in neutralizing pathogenic ANCAs by anti-idiotype antibodies (36, 37). One review in 2013 indicated that IVIG had broad immunosuppression in autoimmune diseases such as the expansion of regulatory T cells, modulation of dendritic cells, and blocking cellular receptors (38). Crickx et al. in 2016 reported a retrospective study with 92 patients with AAV using IVIG including 20 patients with EGPA, and the result supports the use of IVIG in AAV as adjunctive therapy, particularly in relapsing or refractory disease (39). The high-dose IVIG might be effective for patients with heart failure or peripheral neuropathy not responsive to standard immunosuppressive therapy (40–42). A recent study suggested that earlier add-on combination administration of IVIG and mepolizumab might be efficient adjunct treatment to induce clinical remission and decrease relapse risk for patients with EGPA (43).

IVIG does have a certain position in the treatment of EGPA; however, in fact, IVIG is mainly used for critically severe patients in our hospital. First, the cost of IVIG for each course of treatment is 20,000–30,000 yuan and is not covered by public insurance, which is hardly affordable for many patients in China. Second, as a blood product, IVIG sometimes is in a state of insufficient supply. Thus, only patients who have severe heart, CNS, or kidney involvements, or are complicated by severe infection could be administered IVIG. All the above factors may partly explain why IVIG was sometimes not given as the first line in a real-world situation. Hoffmann and Enk in 2019 summarized that although IVIG is generally used as a second- or third-line treatment in autoimmune disease including systemic vasculitis; however, the first-line treatment may be warranted in special conditions like concomitant malignancy, foudroyant clinical courses, and contraindications against alternative treatments (32). For these conditions in EGPA, early IVIG usage might be also beneficial as the first-line therapy, which deserves more trials, especially for those at a high risk of death.

This study has limitations. First, as a tertiary referral center in China, most of enrolled hospitalized patients were relatively complicated or severe cases, and the ANCA-negative patients with EGPA were more likely to be hospitalized, causing the ANCA-positive rate to be relatively lower. Second, considering that the sample size was not large enough, some of the findings did not reach statistical difference but have suggested valuable tendencies. Therefore, we are consecutively enrolling more patients to establish a lager EGPA cohort and the prospective multicenter study is warranted.

In summary, this EGPA cohort from China revealed that the patients with positive MPO-ANCA showed an increased disease activity, higher proportions of renal lesions, and biopsy-proven vasculitis, whereas the patients with negative ANCA had more cardiac involvement and asthma. The titers of MPO-ANCA at baseline correlated positively with disease activity. MPO-ANCA positivity was the most significantly independent factor associated with renal involvement in EGPA.

## Data Availability Statement

The original contributions presented in the study are included in the article/**Supplementary Material**. Further inquiries can be directed to the corresponding authors.

## Ethics Statement

The studies involving human participants were reviewed and approved by ethical committee of Peking Union Medical College Hospital. The patients/participants provided their written informed consent to participate in this study.

## Author Contributions

LW and FZ designed the study. SL analyzed the data and wrote the manuscript. LW revised the manuscript. LH, YL, JY, YZ, and SL collected the data. ML, XT, XZ, LW, and FZ enrolled and managed the patients. All authors contributed to the article and approved the submitted version.

## Funding

The study was supported by the Non-profit Central Research Institute Fund of Chinese Academy of Medical Science (2019XK320022), National Key Research and Development Program (2016YFA0101003 and 2016YFC0903901), National Natural Science Foundation of China (81501414, 81771764, and 81571594), CAMS Innovation Fund for Medical Sciences (2017-I2M-3–008 and 2016-I2M-1–003), and Graduate Innovation Fund of Peking Union Medical College (2019-1002-01).

## Conflict of Interest

The authors declare that the research was conducted in the absence of any commercial or financial relationships that could be construed as a potential conflict of interest.

## Publisher’s Note

All claims expressed in this article are solely those of the authors and do not necessarily represent those of their affiliated organizations, or those of the publisher, the editors and the reviewers. Any product that may be evaluated in this article, or claim that may be made by its manufacturer, is not guaranteed or endorsed by the publisher.

## Supplementary Material

Supplementary Table 1 IVIG usage and outcomes. IVIG, intravenous immunoglobulin.

Supplementary Table 2 Clinical features of EGPA patients who used IVIG. EGPA, eosinophilic granulomatosis with polyangiitis; IVIG, intravenous immunoglobulin; BVAS, Birmingham Vasculitis Activity Score; FFS, five factor score; PNS, peripheral nervous system; Y, yes, means the patients had heart or PNS involvement; N, no, means the patients did not have heart or PNS involvement in the fourth column and means no biologics were used in the “Biologics” column; MP, methylprednisolone; CYC, cyclophosphamide; Pred, prednisone; LEF, leflunomide; MTX, methotrexate; NA, the information was not clear; AZA, azathioprine; MMF, mycophenolate mofetil; CR, complete remission; PR, partial relief. #, the exact dose is unknown.

Supplementary Table 3 Clinical features of deaths in the cohort. BVAS, eosinophilic granulomatosis with polyangiitis; FFS, five factor score; GC, glucocorticoid; CYC, cyclophosphamide; FU, follow-up; Y, yes, means the patients were administered the corresponding drugs; N, no, means the patients were not administered the corresponding drugs; HCQ, hydroxychloroquine; T2, Tripterygium.
