# Supplementary material for: Clinical Significance of MPO-ANCA in Eosinophilic Granulomatosis With Polyangiitis: Experience From a Longitudinal Chinese Cohort
Source: Front Immunol. 2022 Jun 27;13:885198. doi: 10.3389/fimmu.2022.885198 (PMC9271578; doi:10.3389/fimmu.2022.885198)
Supplement: Supplementary Table 1 — IVIG usage and outcomes. IVIG, intravenous immunoglobulin. [file DataSheet_1.docx]

**Supplementary Table 1**. IVIG usage and outcomes

|  | Complete remission | Partial relief | Death |
| --- | --- | --- | --- |
| With IVIG therapy | 17 (81%) | 4 (19%) | 0 (0%) |
| Without IVIG therapy | 73 (76%) | 17 (17.7%) | 6 (6.3%) |

IVIG, intravenous immunoglobulin.

**Supplementary Table 2.** Clinical features of EGPA patients who used IVIG

| Cases | BVAS | FFS | Heart or PNS involvement | Induction therapy | Maintenance therapy | Biologics | Outcomes |
| --- | --- | --- | --- | --- | --- | --- | --- |
| 1 | 11 | 2 | Y | MP 80mg qd + IVIG 10g*4d  + CYC | Pred 10mg qd  + CYC | N | CR |
| 2 | 18 | 0 | N | MP 80mg qd + IVIG 10g*5d  → Pred 60mg qd + CYC | Pred 7.5mg qd | N | CR |
| 3 | 15 | 2 | Y | MP pulse + IVIG 25g*5d  + CYC | Pred 12.5mg qd + MTX | N | CR |
| 4 | 17 | 3 | Y | MP pulse + IVIG 10g*3d  + CYC | Pred 10mg qd  + CYC | N | PR |
| 5 | 18 | 1 | Y | MP pulse + IVIG 20g*5d  + CYC | Pred 10mg qd  + MTX | N | CR |
| 6 | 19 | 1 | N | MP 80mg qd + IVIG 4d^#^  + CYC | Pred 15mg qd  + CYC | N | CR |
| 7 | 10 | 1 | Y | MP 250mg qd + IVIG 10g*3d  + CYC | NA | N | PR |
| 8 | 20 | 2 | Y | MP pulse + IVIG 20g*3d  + CYC | NA | N | CR |
| 9 | 20 | 2 | Y | MP 80mg qd + IVIG 20g*5d  + CYC | Pre 10mg qod  + MTX | N | PR |
| 10 | 26 | 3 | Y | MP pulse + IVIG 20g*5d  + CYC | Pred 10mg qd  + CYC | N | CR |
| 11 | 19 | 0 | Y | MP pulse + IVIG 20g*3d  + CYC + plasma exchange | Pred 10mg qd  + CYC | N | CR |
| 12 | 15 | 1 | Y | MP 60mg qd + CYC  + IVIG 20g*3d | Pred 2.5mg qd + AZA | N | CR |
| 13 | 24 | 1 | Y | MP 80mg qd + LEF + IVIG  20g*3d→Pred 60 mg qd + CYC | Pred 5mg qd  + CYC + LEF | N | CR |
| 14 | 30 | 2 | Y | MP 80mg qd + IVIG 20g*5d  + CYC | MP 2mg qd | N | CR |
| 15 | 41 | 2 | Y | MP 80mg qd + IVIG 20g*5d  + CYC | MTX | N | CR |
| 16 | 29 | 1 | Y | MP 0.2g qd + plasma exchange  + CYC + IVIG 20g*3d | No drugs | N | PR |
| 17 | 17 | 1 | Y | MP 80mg qd + IVIG 20g*3d  + CYC→ MP pulse + CYC | Pred 10mg qod  + MMF | N | CR |
| 18 | 18 | 0 | Y | MP 80mg qd + IVIG^#^ + CYC | Pred 10mg qd  + LEF | N | CR |
| 19 | 6 | 2 | N | IVIG 20g *5d + MP 160mg qd  + CYC | No drugs | N | CR |
| 20 | 18 | 3 | Y | MP 160mg qd + IVIG^#^ + CYC | Pred^#^ + CYC | N | CR |
| 21 | 6 | 2 | N | IVIG 20g *5d + MP 80mg qd  + CYC + MTX | Pred 5mg qd  + AZA | N | CR |

EGPA, eosinophilic granulomatosis with polyangiitis; IVIG, intravenous immunoglobulin; BVAS, Birmingham Vasculitis Activity Score; FFS, five factor score; PNS, peripheral nervous system; Y, yes, means the patients had heart or PNS involvement; N, no, means the patients did not have heart or PNS involvement in the fourth column and means no biologics were used in the “Biologics” column; MP, methylprednisolone; CYC, cyclophosphamide; Pred, prednisone; LEF, leflunomide; MTX, methotrexate; NA, the information was not clear; AZA, azathioprine; MMF, mycophenolate mofetil; CR, complete remission; PR, partial relief. #, the exact dose is unknown.

**Supplementary Table 3.** Clinical features of deaths in the cohort

| Case | BVAS | FFS | GC | CYC | Biologics | Other immune-suppressants | FU time, months | Cause of death |
| --- | --- | --- | --- | --- | --- | --- | --- | --- |
| 1 | 22 | 0 | Y | Y | N | N | 1 | Small cell lung cancer |
| 2 | 27 | 1 | Y | Y | N | N | 1 | Digestive tract perforation |
| 3 | 23 | 1 | Y | Y | N | N | 12 | Chronic myelomonocytic leukemia |
| 4 | 40 | 2 | Y | Y | N | N | 2 | Intestinal perforation with infection and myocardial involvement |
| 5 | 9 | 3 | Y | N | N | HCQ+T2 | 6 | Multiple organ failure with severe infection |
| 6 | 11 | 1 | Y | Y | N | N | 4 | Sudden aneurysm rupture |

BVAS, eosinophilic granulomatosis with polyangiitis; FFS, five factor score; GC, glucocorticoid; CYC, cyclophosphamide; FU, follow-up; Y, yes, means the patients were administered the corresponding drugs; N, no, means the patients were not administered the corresponding drugs; HCQ, hydroxychloroquine; T2, Tripterygium.
